# Microwave photonics doppler speed measurement based on sagnac loops and four-wave mixing effect in a highly nonlinear fiber

**DOI:** 10.1038/s41598-024-56470-y

**Published:** 2024-03-08

**Authors:** Hossein Emami, Reza Hashemi

**Affiliations:** 1 Department of Skill Development and Entrepreneurship, Isfahan (Khorasgan) Branch, Islamic Azad University, Isfahan, Iran; 2 Department of Computer Science, San Diego State University, San Diego, CA 32611 USA

**Keywords:** Electrical and electronic engineering, Applied optics

## Abstract

Photonic radars are increasingly being developed and offer a promising replacement for traditional RF radars. They feature higher precision, and smaller size compared to the current microwave radars. One important part of a moving target indicating (MTI) radar is the Doppler shift measurement used to measure the radial velocity of a moving target. Therefore, for any photonic radar operating at MTI mode, it is necessary to have a Doppler measurement subsystem. In this paper, a microwave photonic Doppler frequency measurement system is conceived and implemented for this purpose specifically. The operation is based on making a Doppler shift-dependent yet low-frequency voltage component. It is all-optical and hence has the potential to be integrated into many electronic warfare systems. This feature not only makes the system independent of any sophisticated electrical device but also makes the measurement time lower than that of the electrical counterparts. The specific design presented here provides a much better stability compared to the recent works. An error as low as 0.012 Hz at a 10 GHz radar frequency was obtained, and the system performance was demonstrated up to 40 GHz, at which a 4.75 Hz error was recorded.

## Introduction

Doppler shift is a physical phenomenon that has various applications in science and engineering. Some examples are sonography^1–4^, astronomy^5–8^, and vibrometry^9–13^. One traditional yet essential application is the moving target indication (MTI) radar, where Doppler shift is utilized to estimate the target speed^14,17^. In recent years, photonic radars have attracted much attention^18–24^ owing to the distinctive features of photonics technology over traditional microwave engineering^25–28^, making the integration of Doppler measurement in this radar category inevitable. Several endeavors have been undertaken to implement such microwave-photonics Doppler frequency measurement^29–36^. These implementations were all grounded on the downconversion of the radar signal on which the Doppler shift is modulated. The downconverting process was performed by a single or dual Mach‒Zehnder modulator. The baseband signal frequency was then measured using an electrical signal analyzer (ESA) and/or an oscilloscope, and therefore, the systems were capable of providing extremely accurate measurements of Doppler frequency. Additionally, the latency would be mainly determined by the ESA since it has to scan a band to acquire the Doppler frequency. Specifically, obtaining higher resolutions requires a slower scanning process. The MZMs in the system were intensity modulators and thus needed bias controllers to ensure stability.

Our group has developed a class of Doppler frequency measurement systems based on Doppler frequency-to-voltage mapping^37^. In our design, no sophisticated electrical equipment nor devices were used, and only a DC voltmeter was used, leading to extremely lower the cost of the entire system. The measurement latency was also improved, as no frequency band scanning was needed, and it was merely limited by the voltmeter latency, which was constant at all resolutions and frequencies. It could operate over a broad radar frequency range with excellent accuracy.

In this paper, we propose a new category of Doppler frequency measurement systems based on phase modulators within parallel Sagnac loops that do not need any bias control. This would enhance the stability of the system and thus the complexity. On the other hand, this design provides much better accuracy without sacrificing other crucial characteristics such as latency, etc. We achieved an estimation error of 0.15 km/h at a radar frequency of 40 GHz for objects with a maximum speed of Mach 10. This broadband operation capability has also made it an excellent candidate for frequency agility systems.

## Results

### Experimental setup

**Figure 1:**
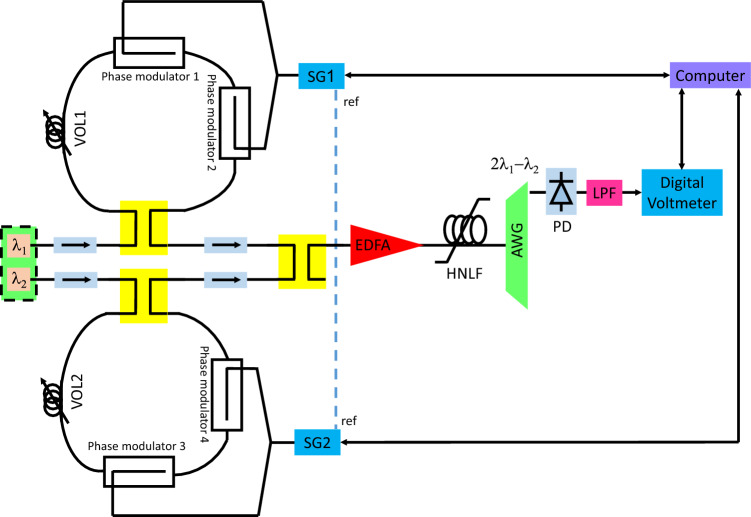
Experimental setup of the MWP Doppler frequency measurement system.

Figure 1 depicts an experimental setup for Doppler frequency measurement purposes. A laser array provided two optical carriers with wavelengths λ_1_ and λ_2_. Each optical carrier fed a Sagnac loop via an optical isolator and a 3-dB optical coupler. Within each Sagnac loop, two phase modulators (LN27S-FC, Thorlabs) were placed. Variable optical lengths (VOLs) were also placed within the loop. The upper loop was fed by an RF tone produced by an RF signal generator (SG1, MG3694B, Anritsu) with an angular frequency of Ω + Ω_*d,*_ and the lower loop was fed by another RF signal generator (SG2) with an RF tone with angular frequency Ω. Ω and Ω_*d*_ denote the radar and Doppler frequencies, respectively. The SG1 reference output was connected to the SG2 reference input. Wilkinson power dividers were used to split the RF tones evenly feeding each pair of modulators in each loop. A 3-dB optical coupler combined the loop outputs via optical isolators. The coupler output was amplified by an erbium-doped fiber amplifier (EDFA, EDFA300P, Thorlabs) and then traversed through a highly nonlinear fiber (HLNF, PMHN3N, Thorlabs), and the result was filtered at 2λ_2_ − λ_1_ by an arrayed waveguide grating (AWG, FFC-MUX-017D, JDSU). The AWG output was then detected by a photodetector (PD, XPDV 2020R, u^2^t photonics). A low-pass filter extracted the low-frequency component of the PD output, and finally, a digital voltmeter measured this component. A desktop computer was employed to control SG1, SG2, and the digital voltmeter.

### Theoretical analysis

Having conceived a microwave photonic system to measure Doppler frequency, we must now mathematically model the system output. We thus begin with the optical carrier whose electric fields can be written as $E_{1} = P_{ \circ }^{\frac{1}{2}} e^{{j\omega_{1} t}}$ and $E_{1} = P_{ \circ }^{\frac{1}{2}} e^{{j\omega_{2} t}}$ where *P*_o_, *ω*_1_, and *ω*_2_ are the optical power of each carrier at the loop input and angular frequencies of λ_1_ and λ_2_, respectively. Each carrier was divided into two equal portions within the optical couplers. One portion traversed through the loop clockwise, and the other traversed in a counterclockwise direction. Each portion was modulated by the RF tones generated by the signal generators within a phase modulator. Both portions were combined at the other side of the loops by 3-dB optical couplers. The output of the upper and lower couplers can be described as $E_{U} = E_{U,CW} + E_{U,CCW}$ $E_{L} = E_{L,CW} + E_{L,CCW}$ where indices U, L, CW, and CCW indicate the upper loop, lower loop, clockwise, and counterclockwise, respectively. Using the same method as^38^, E_*U*_ and E_*L*_ can be calculated as:

1 $$E_{U} = \left( {\frac{{L_{1} L_{2} }}{4}} \right)^{\frac{1}{4}} P_{ \circ }^{\frac{1}{2}} e^{{j\omega_{1} t}} \left( {e^{{j\beta_{1} \cos \left[ {(\Omega + \Omega_{d} )(t + \tau )} \right]}} - e^{{j\beta_{2} \cos (\Omega + \Omega_{d} )t}} } \right),\quad E_{L} = \left( {\frac{{L_{3} L_{4} }}{4}} \right)^{\frac{1}{4}} P_{ \circ }^{\frac{1}{2}} e^{{j\omega_{2} t}} \left( {e^{{j\beta_{3} \cos \Omega (t + \tau )}} - e^{{j\beta_{4} \cos \Omega t}} } \right)$$

where *L*_*i*_ and *β*_*i*_ (i = 1,…,4) denote the optical insertion loss and modulation index of each modulator. The modulation index *β*_*i*_ can be expressed as $\beta_{i} = \frac{\pi }{{V_{{\pi_{i} }} }}\left( {MP_{RF} Z_{RF} } \right)^{\frac{1}{2}}$ where $V_{{\pi_{i} }}$(i = 1,…,4) denotes the half-wave voltage of each modulator. *M* and *Z*_*RF*_ are the RF response, and input impedance, respectively.

The delay *τ* is defined as

2 $$\tau = \frac{n\Delta L}{c}$$

where *n* is the fiber refractive index and Δ*L* is phase modulator 2 and 3 distance from the loop center (the same distance was assumed for modulators 2 and 3). *c* is the speed of light in the vacuum.

Expanding *E*_*U*_ and *E*_*L*_ in terms of Fourier series, ignoring all components except the first one, and assuming small signal conditions for all modulators, we have:

3 $$\begin{gathered} E_{U} = j\left( {\frac{{L_{1} L_{2} }}{4}} \right)^{\frac{1}{4}} P_{ \circ }^{\frac{1}{2}} e^{{j\omega_{1} t}} \left[ {\beta_{1} \cos \left[ {(\Omega + \Omega_{d} )(t + \tau )} \right] - \beta_{2} \cos (\Omega + \Omega_{d} )t} \right], \hfill \\ \quad \quad \quad \quad \quad \quad \quad \quad \quad \quad \quad \quad \quad \quad \quad \quad \quad \quad E_{L} = j\left( {\frac{{L_{3} L_{4} }}{4}} \right)^{\frac{1}{4}} P_{ \circ }^{\frac{1}{2}} e^{{j\omega_{2} t}} \left( {\beta_{3} \cos \Omega (t + \tau ) - \beta_{4} \cos \Omega t} \right) \hfill \\ \end{gathered}$$

modulator, respectively. *P*_*RF*_ denotes the RF power output of SG1 and SG2 (the same RF power for both were assumed. The electric fields were combined with a 3-dB optical coupler and were amplified by the EDFA. The EDFA output entered the HLNF and experienced the our-wave mixing effect. As a result, several harmonics were generated from which we are interested in 2*ω*_2_-*ω*_1_. This harmonic was separated from the others using the AWG, and it can be calculated using the method of^37^:

4 $$\begin{gathered} E_{{2\omega_{2} - \omega_{1} }} = G^{\frac{3}{2}} \left( {\frac{{L_{1} L_{2} L_{3}^{2} L_{4}^{2} }}{64}} \right)^{\frac{1}{4}} G_{FWM}^{\frac{1}{2}} L_{AWG}^{\frac{1}{2}} P_{ \circ }^{\frac{3}{2}} e^{{j(2\omega_{2} - \omega_{1} )t}} \hfill \\ \quad \quad \quad \quad \quad \times \left[ {\beta_{1} \cos \left[ {(\Omega + \Omega_{d} )(t + \tau )} \right] - \beta_{2} \cos (\Omega + \Omega_{d} )t} \right]\left( {\beta_{3} \cos \Omega (t + \tau ) - \beta_{4} \cos \Omega t} \right)^{2} \hfill \\ \end{gathered}$$

where *G*, *G*_*FWM*_, and *L*_*AWG*_ denote the EDFA gain, FWM conversion gain, and AWG insertion loss, respectively. This field is then detected by the PD whose output current would be $I = \Re \left\| {E_{{2\omega_{2} - \omega_{1} }} } \right\|^{2}$ where $\Re$ is the PD responsivity. This current established a voltage whose DC component was separated by the LPF and was measured by the digital voltmeter. This voltage can be written as:

5 $$\begin{gathered} V_{DC} = \frac{3}{64}\Re G_{LPF} Z_{LPF} G^{3} \left( {L_{1} L_{2} } \right)^{\frac{1}{2}} L_{3} L_{4} G_{FWM} L_{AWG} P_{ \circ }^{3} \hfill \\ \quad \quad \quad \times \left[ \begin{gathered} \left( {\beta_{1}^{2} + \beta_{2}^{2} } \right)\left( {\beta_{3}^{4} + \beta_{4}^{4} + 4\beta_{3}^{2} \beta_{4}^{2} } \right) - 2\left[ {\left( {\beta_{1}^{2} + \beta_{2}^{2} } \right)\beta_{3} \beta_{4} \left( {\beta_{3}^{2} + \beta_{4}^{2} } \right) + \beta_{1} \beta_{2} \left( {\beta_{3}^{4} + \beta_{4}^{4} + 5\beta_{3}^{2} \beta_{4}^{2} } \right)} \right]\cos \Omega \tau \hfill \\ + 4\beta_{1} \beta_{2} \beta_{3} \beta_{4} \left( {\beta_{3}^{2} + \beta_{4}^{2} } \right)\cos \Omega_{d} \tau - 2\beta_{1} \beta_{2} \beta_{3}^{2} \beta_{4}^{2} \cos 3\Omega \tau + 2\beta_{3}^{{}} \beta_{4}^{{}} \left[ {2\beta_{1} \beta_{2} \left( {\beta_{3}^{2} + \beta_{4}^{2} } \right) + \beta_{3}^{{}} \beta_{4}^{{}} \left( {\beta_{1}^{2} + \beta_{2}^{2} } \right)} \right]\cos 2\Omega \tau \hfill \\ \end{gathered} \right] \hfill \\ \end{gathered}$$

where *Z*_*LPF*_ and *G*_*LPF*_ denote the LPF input impedance and gain, respectively. It can be seen that the only unknown parameter in Eq. (5) is the Doppler frequency Ω_*d*_; thus, by measuring *V*_*DC*_, Ω_*d*_ can be identified. Note that since cosΩ_*d*_*τ* is a periodic function, the system is able to identify the Doppler frequencies within a half-period. Furthermore, in real applications, low Doppler frequencies are often of interest; thus, we consider Doppler frequencies from 0 to 1/2*τ*. Since $f_{d} = \frac{V}{c}f_{c}$, this will result in measuring a target speed of 0 to $V_{\max } = \frac{c}{{2\tau f_{c} }}$. It can be seen that the maximum target speed is inversely related to the delay *τ* and the radar carrier frequency. Therefore, a higher radar frequency will result in a lower maximum speed measurement. Here, we consider a worst-case scenario of *f*_*c*_ = 40 GHz since the modulators cannot operate beyond this frequency. Note that in the real EW environment, the fastest target to be detected would be the zircon missile with Mach 8 speed^39^. We thus consider 10 Mach speed to cover this. The delay *τ* is thus calculated to be *τ* = 1.0965 *μ*sec. From Eq. (2), Δ*L* can be calculated as Δ*L*≈225.6 m given a refractive index of *n* = 1.458. This Δ*L* must be precisely applied to both loops. This was performed by careful adjustment of VOLs.

### Spectrum analysis

**Figure 2:**
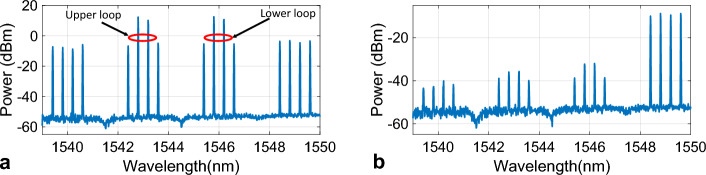
The spectrum at the (**a**) HNLF output, (**b**) AWG output.

An optical spectrum analyzer was used to observe the HNLF output where both SG1, and SG2 frequencies were set to 10 GHz (Ω_*d*_ = 0). Figure 2a shows the spectrum at the HLNF output for a 25 GHz RF frequency. Many harmonics were generated by four-wave mixing, as expected. Note that each loop generated a double-sideband suppressed carrier modulation. Thus, the input spectrum consisted of two pairs of carriers each generated by a loop, as shown in Fig. 2a. The AWG filtered all harmonics except the one at 2*ω*_2_-*ω*_1,_ as depicted in Fig. 2b. It can be seen that four wavelengths at 1548.4, 1548.8, 1549.2, and 1549.6 nm were separated from the others. In the absence of Doppler frequency (Ω_*d*_ = 0), all carriers have the same amplitude as predicted by Eq. (3). Calibration was required due to additional loss made by fiber patch cords and connectors and it was performed to match the output voltage with Eq. (5). This was accomplished by introducing a calibration factor (*C*_*f*_) to Eq. (5) as a single coefficient as below:

6 $$V_{DC\_Cal} = C_{f} V_{DC}$$

*C*_*f*_ was measured to be 0.92.

### Doppler shift measurement

In the next step, we began introducing the Doppler frequency into the upper loop.

**Figure 3:**
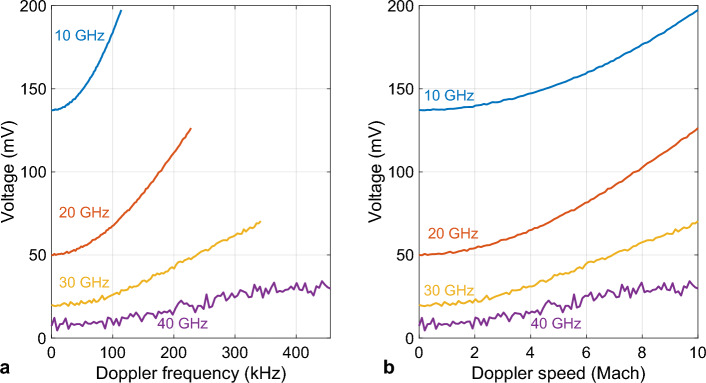
Output voltage at various RF frequencies versus (**a**) Doppler frequency, and (**b**) Doppler speed.

The results are shown in Fig. 3a. It can be seen that the voltage curves have an ascending behavior with Doppler frequency increase. However, at lower RF frequencies, the curves are less noisy. This is due to the RF response of the modulators, which has descending characteristics with increasing frequency. Specifically, at 40 GHz, the voltage curve has a higher noise level compared to lower frequencies which could cause a larger error in the speed estimation procedure. This can be attributed to the 35 GHz 3-dB bandwidth of the modulators.

Moreover, there can be seen an offset for each curve that decreases with RF frequency increments, which was expected from Eq. (5). The voltage has an RF frequency-dependent part that has a descending response with the RF frequency. In addition, it can be seen that at low RF frequencies, the voltage curves have a sharper slope thus it can be inferred that Doppler measurement will be more accurate at lower RF frequencies.

**Figure 4:**
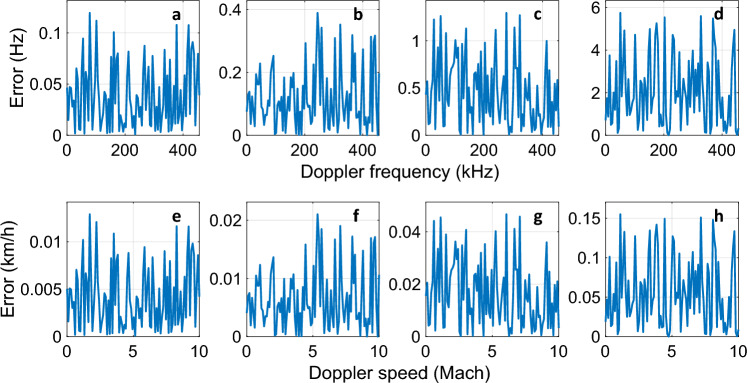
Measurement error for carrier frequencies of (**a**,**e**) 10, (**b**,**f**) 20, (**c**,**g**) 30, and (**d**,**h**) 40 GHz. The first row shows error for Doppler frequency and the second row shows error for Doppler speed.

For practical purposes, the results of Fig. 3a were employed to build Fig. 3b, which shows the output voltage as a function of Doppler speed. It can be seen the identification of the Doppler speed would be possible within a range of 0 to Mach 10 at all RF frequencies, although it could be challenging at 40 GHz due to the high noise level, as discussed before. To investigate this further, the measurement error for Doppler frequency was calculated at each RF frequency, and the results are provided in Fig. 4a–d. As expected, at 10 GHz, the lowest measurement error was achieved (a maximum of 0.012 Hz). At 20, 30, and 40 GHz, the error increases with frequency increment (0.39, 1.29, and 5.75 Hz, respectively). These results were used to extract the Doppler speed error based on $\Delta V = \frac{c}{{f_{c} }}\Delta f_{d}$, and the results are depicted in Fig. 4e–h. The maximum error at 10, 20, and 30 GHz were 0.013, 0.021, and 0.047 km/h, respectively. At 40 GHz, the maximum error exceeds 0.1 km/h and becomes 0.15 km/h.

## Discussion

The system can identify the speed of moving objects up to Mach 10 over a wide radar frequency of up to 40 GHz with 0.15 km/h error. Note that specific values for speed measurement error in radars can vary widely depending on the application the radar is designed for as well as the specific conditions the radar operates in. While it is challenging to provide an exact number for each system feature, it would be possible to compare the system characteristics to those of the others in order to evaluate the system operation. Table 1 compares the characteristics of this work with previous attempts. It is evident that our system is the fastest one. Regarding the measurement error, although^33–36^ provided a smaller error, this was at the cost of extremely high latency. Specifically, the error was very low in^34^; however, the latency was 40 s. The resolution of our system was also lower than the others except^37^ which exhibits an error as high as 650 times the present work. In summary, our system exhibits the best latency as well as acceptable or better performance in the other aspects listed in Table 1.

**Table 1:** Comparison of the present work and the other attempts.

	^23^	^24^	^29^	^30^	^31^	^32^	^33^	^34^	^35^	^36^	^37^	^41^	This work
Error (km/h)	2	2.7	0.22	225	0.54	NP	.0032	0.027	0.13	0.06	98.5	615	0.15
Latency (ms)	< 20	NP	NP	NP	250 s	NP	50 s	40 s	239s	60 s	< 2	1	0.4
Maximum radar frequency (GHz)	40	11.2	15.2	24	12	35	16.1	20	40	18	40	40	40
ESA/OSC required	N	Y	Y	Y	Y	Y	Y	Y	Y	Y	N	N	N
Speed measurement range (Mach)	NP	NP	2.88	7.31	7.31	10	5.45	4.39	2.19	4.87	10	10	10
Demonstrated Resolution (Mach)	NP	NP	0.58	NP	.73	NP	0.55	0.44	0.22	0.49	0.0022	NP	0.0022

*NP: Not provided.

At higher frequencies, the measurement error tends to rise. Nevertheless, the error could be reduced by choosing a smaller maximum value for the Doppler frequency. In this case, the system RF range will increase. The other solution is to employ modulators with higher cutoff frequencies^40^; nonetheless, the cost of the system will increase. Another alternative is to conduct a relatively large number of measurements simultaneously. This will decrease the noise level, thereby increasing measurement accuracy. However, this will be at the cost of higher latency, which could or could not be a problem depending on the application for which the system is designed. In applications that require low latency, the system could first perform a rough but fast measurement, and then a more accurate but slower measurement will be conducted by averaging a series of independent measurements.

Another approach toward the accuracy increase issue is to employ lock-in amplification to reduce the noise. In this technique, the term to be measured (cos Ω_*d*_*τ* in Eq. (5)) is separated from the other terms. This approach allows obtaining a more accurate value for the output voltage^41^. Nevertheless, this will also be at the cost of higher latency.

The system operation is all-optical, and there is no need for any high-frequency electrical measurement equipment. Frequency agile systems could also benefit from this system since it can operate over a broad carrier frequency range. For frequency agility purposes a more rigorous characterization might be required. This will include system characterization at all carriers at which the frequency hopping will be happening.

Since no intensity modulator has been used, the system operation will not be affected by the modulator bias drifts and this could provide better stability. This was obvious during the measurement procedure as we could manage to run the experiment several times without the need to re-bias the modulators. The HNLF used to generate FWM can be replaced by nonlinear photonic crystals^42^. This would make system integration possible and additionally will further improve the stability of the system.

Using FWM would degrade the efficiency of the system, as the conversion gain of FWM is usually low. That is the reason an EDFA is required before the HNLF. The sensitivity degradation is however overridden by using phase modulators in Sagnac loops. The explanation is as follows.

Equation (3) shows that all input components to the HNLF are frequency-dependent and there is no large constant (frequency-independent) term that would have consequently made a large frequency-independent term at the HNLF output. This constant component (sometimes 10^4^ larger than the frequency-dependent one caused by intensity modulators operating at quadrature bias) makes the voltmeter saturated and as a result, the voltmeter has to operate in a course mode to avoid saturation. This will ultimately result in less accurate measurement even without any FWM component in the system. On the other hand, in this design from Eq. (5) it can be seen that the constant term is of the same order of amplitude compared to the frequency-dependent term and thus causes an extremely smaller contribution in the voltage measurement error; therefore, the voltmeter can provide much better precision in measuring the frequency-dependent term. This is also evident from Fig. 3 in which in the worst case the ratio of the frequency-dependent term to the frequency-independent term is only 0.5.

We have demonstrated practically that the system of Fig. 1 can measure Doppler shifts with a continuous wave (CW) single tone. Here we theoretically demonstrate its feasibility for the case of linear frequency modulated (LFM) CW radar.

In the case of LFM radar, the values of *E*_*U*_, and *E*_*L*_ for a single cycle can be written as:

7 $$\begin{gathered} E_{U} = j\left( {\frac{{L_{1} L_{2} }}{2}} \right)^{\frac{1}{4}} P_{ \circ }^{\frac{1}{2}} e^{{j\omega_{1} t}} \left[ {\beta_{1} \cos \left[ {(\Omega + \Omega_{d} )(t + \tau ) + \pi K_{^\circ } (t + \tau )^{2} } \right] - \beta_{2} \cos \left[ {(\Omega + \Omega_{d} )t + \pi K_{^\circ } t^{2} } \right]} \right],\quad \hfill \\ E_{L} = j\left( {\frac{{L_{3} L_{4} }}{2}} \right)^{\frac{1}{4}} P_{ \circ }^{\frac{1}{2}} e^{{j\omega_{2} t}} \left[ {\beta_{3} \cos \left( {\Omega (t + \tau )\pi K_{^\circ } (t + \tau )^{2} } \right) - \beta_{4} \cos \left( {\Omega t + \pi K_{^\circ } t^{2} } \right)} \right] \hfill \\ \end{gathered}$$

where *K*_o_ (*K*_o_ ≠ 0) is the chirp rate of the radar []. Thus, the AWG output electric field can be written as:

8 $$\begin{gathered} E_{{2\omega_{2} - \omega_{1} }} = - jG^{\frac{3}{2}} \left( {\frac{{L_{1} L_{2} L_{3}^{2} L_{4}^{2} }}{64}} \right)^{\frac{1}{4}} G_{FWM}^{\frac{1}{2}} L_{AWG}^{\frac{1}{2}} P_{ \circ }^{\frac{3}{2}} e^{{j(2\omega_{2} - \omega_{1} )t}} \times \hfill \\ \quad \quad \quad \quad \quad \quad \quad \quad \quad \left[ {\beta_{1} \cos \left[ {(\Omega + \Omega_{d} )(t + \tau )} \right] - \beta_{2} \cos (\Omega + \Omega_{d} )t} \right]\left( {\beta_{3} \cos \Omega (t + \tau ) - \beta_{4} \cos \Omega t} \right)^{2} \hfill \\ \end{gathered}$$

Consequently, the output voltage will be:

9 $$V_{DC} = \frac{3}{64}\Re G_{LPF} Z_{LPF} G^{3} \left( {L_{1} L_{2} } \right)^{\frac{1}{2}} L_{3} L_{4} G_{FWM} L_{AWG} P_{ \circ }^{3} \left[ {\left( {\beta_{1}^{2} + \beta_{2}^{2} } \right)\left( {\beta_{3}^{4} + \beta_{4}^{4} + 4\beta_{3}^{2} \beta_{4}^{2} } \right) + 4\beta_{1} \beta_{2} \beta_{3} \beta_{4} \left( {\beta_{3}^{2} + \beta_{4}^{2} } \right)\cos \Omega_{d} \tau } \right]$$

This voltage is for a single cycle for LFM radar. Assuming a radar duty cycle of *D*, the output voltage will become[tim]:

10 $$\begin{gathered} V_{DC} = \frac{3}{64}\Re DG_{LPF} Z_{LPF} G^{3} \left( {L_{1} L_{2} } \right)^{\frac{1}{2}} L_{3} L_{4} G_{FWM} L_{AWG} P_{ \circ }^{3} \times \hfill \\ \quad \quad \quad \quad \quad \quad \quad \quad \quad \quad \quad \quad \left[ {\left( {\beta_{1}^{2} + \beta_{2}^{2} } \right)\left( {\beta_{3}^{4} + \beta_{4}^{4} + 4\beta_{3}^{2} \beta_{4}^{2} } \right) + 4\beta_{1} \beta_{2} \beta_{3} \beta_{4} \left( {\beta_{3}^{2} + \beta_{4}^{2} } \right)\cos \Omega_{d} \tau } \right] \hfill \\ \end{gathered}$$

It can be seen from Eq. (10) that in the case of LFM radar, again the only unknown value is Ω_*d*_, thus the system should be able to identify the Doppler shift in this case, too.

## Conclusions

We have demonstrated a Doppler speed measurement system practically. The system exhibits measurement error as low as 0.15 km/h yet can operate over a broad frequency range of up to 40 GHz. The demonstration is all-optical and has the potential to be integrated as a subsystem for many hybrid or all-optical electronic warfare systems that need to measure the Doppler shift. Utilization of phase modulators instead of intensity modulators would provide better stability and less complexity at the same time since there is no need for any bias control circuit.

## Methods

### System configuration for spectrum analysis

The system was configured as depicted in Fig. 1. Wavelengths λ_1_ and λ_2_ were set to 1543 and 1546 nm, respectively. Each laser power was initially set such that it exhibited 10 dBm at the relevant loop entrance. *Z*_*LPF*_, and *Z*_*RF*_ were 1 kΩ, and 50 Ω, respectively. *G*, *G*_*FWM*_, *L*_*AWG*_, and *L* were 20, − 18, − 2, and − 4 dB, respectively. *L*_1_ − *L*_4_ were measured to be − 3.9, − 3.8, − 4.2, and − 4 dB respectively. $\Re$, and *M* were frequency-dependent and were extracted from the datasheets^43,44^. *V*_π1_ − *V*_π4_ were measured to be 7.1, 7.4, 8, and 7.2 V, respectively. To ensure identical Δ*L* for both loops, both signal generators were initially set to 10 GHz and 10 dBm output power. This led to Ω_*d*_ = 0. The VOLs were then adjusted to obtain the maximum output voltage (cosΩ_*d*_*τ* = 1). The laser powers were monitored at the HLNF output, and further adjustments were performed via power adjustments of λ_1_ and λ_2_ to obtain the same amplitude for both wavelengths.

### System configuration for Doppler speed measurement

The frequency was swept by 100 Hz steps from zero to the maximum Doppler frequency equivalent to Mach 10 speed at various RF frequencies (10, 20, 30, and 40 GHz). Table 2 shows these frequencies. At each step, the voltage was read by the voltmeter and was recorded into the computer.

**Table 2:** RF frequencies and the relevant maximum Doppler frequencies.

RF frequency (GHz)	Mach 10 equivalent maximum Doppler frequency (kHz)
10	114
20	228
30	342
40	456

## Data Availability

The datasets used and/or analyzed during the current study are available from the corresponding author upon reasonable request.
